# Supplementary material for: Splice-Junction-Based Mapping of Alternative Isoforms in the Human Proteome
Source: Cell Rep. Author manuscript; Available in PMC 2020 Jan 15. (PMC6961840; doi:10.1016/j.celrep.2019.11.026)
Supplement: 2 [file NIHMS1546469-supplement-2.zip › DF1/PXD009737/TestisTrypsin/TestisTrypsin_12_KIAA1210_NLQVIVEPKEEEPNLPLVSEEEK.pdf]

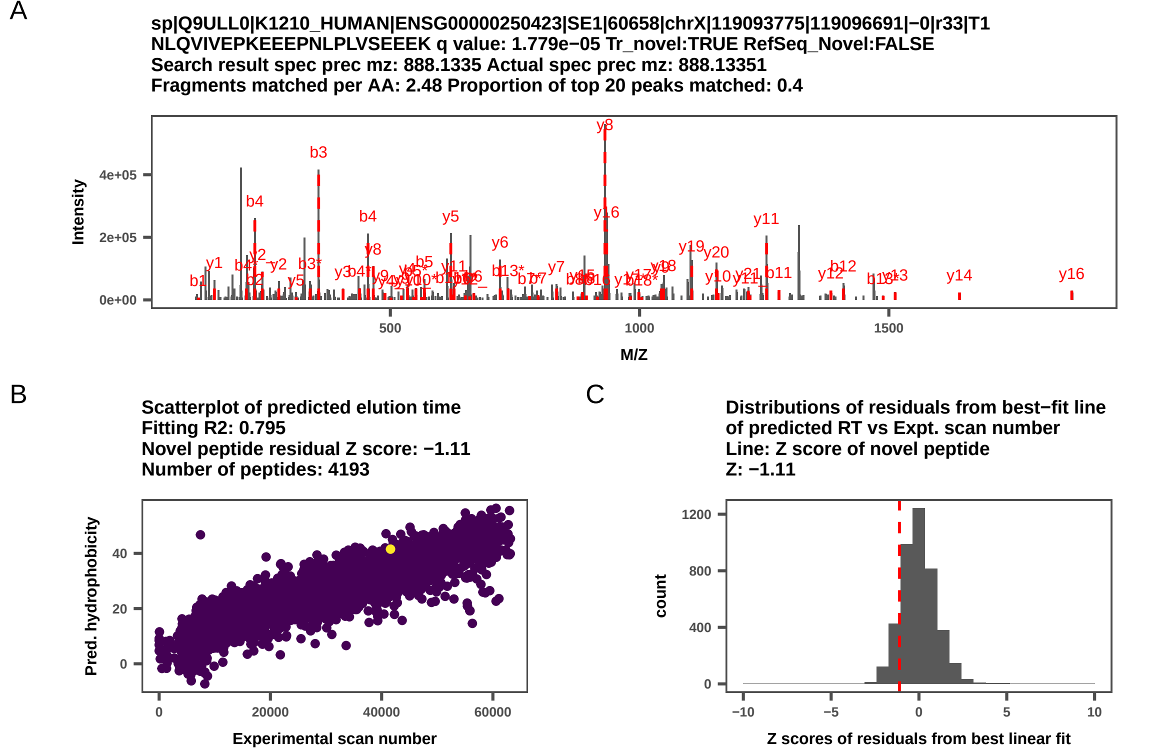
A
sp|Q9ULL0|K1210_HUMAN|ENSG00000250423|SE1|60658|chrX|119093775|119096691|−0|r33|T1
NLQVIVEPKEEEPNLPLVSEEEK q value: 1.779e−05 Tr_novel:TRUE RefSeq_Novel:FALSE
Search result spec prec mz: 888.1335 Actual spec prec mz: 888.13351
Fragments matched per AA: 2.48 Proportion of top 20 peaks matched: 0.4
Intensity
4e+05
2e+05
0e+00
M/Z
500
1000
1500
y8
b3
b4
y16
y11
y5
y6
y19
y20
y14
y16
B
Scatterplot of predicted elution time
Fitting R2: 0.795
Novel peptide residual Z score: −1.11
Number of peptides: 4193
Pred. hydrophobicity
40
20
0
Experimental scan number
0
20000
40000
60000
C
Distributions of residuals from best−fit line
of predicted RT vs Expt. scan number
Line: Z score of novel peptide
Z: −1.11
count
1200
800
400
0
Z scores of residuals from best linear fit
−10
−5
0
5
10
